# Evaluation of acrylamide-based molecularly imprinted polymer thin-sheets for specific protein capture—a myoglobin model

**DOI:** 10.1088/2057-1976/ac0991

**Published:** 2021-06-18

**Authors:** Mark V Sullivan, Sarah R Dennison, Joseph M Hayes, Subrayal M Reddy

**Affiliations:** 1 Dr. M. V. Sullivan and Prof. S. M. Reddy, Department of Chemistry, School of Natural Sciences, University of Central Lancashire, Preston, PR1 2HE, United Kingdom; 2 Dr. S. R. Dennison and Dr. J. M. Hayes, School of Pharmacy and Biomedical Sciences, University of Central Lancashire, Preston, PR1 2HE, United Kingdom

**Keywords:** molecularly imprinted polymer, FTIR spectroscopy, protein, hydrogel, thin-sheet MIP

## Abstract

We evaluate a series of thin-sheet hydrogel molecularly imprinted polymers (MIPs), using a family of acrylamide-based monomers, selective for the target protein myoglobin (Mb). The simple production of the thin-sheet MIP offers an alternative biorecognition surface that is robust, stable and uniform, and has the potential to be adapted for biosensor applications. The MIP containing the functional monomer *N*-hydroxymethylacrylamide (NHMAm), produced optimal specific rebinding of the target protein (Mb) with 84.9% (± 0.7) rebinding and imprinting and selectivity factors of 1.41 and 1.55, respectively. The least optimal performing MIP contained the functional monomer *N,N*-dimethylacrylamide (DMAm) with 67.5% (± 0.7) rebinding and imprinting and selectivity factors of 1.11 and 1.32, respectively. Hydrogen bonding effects, within a protein-MIP complex, were investigated using computational methods and Fourier transform infrared (FTIR) spectroscopy. The quantum mechanical calculations predictions of a red shift of the monomer carbonyl peak is borne-out within FTIR spectra, with three of the MIPs, acrylamide, N-(hydroxymethyl) acrylamide, and *N*-(hydroxyethyl) acrylamide, showing peak downshifts of 4, 11, and 8 cm^−1^, respectively.

## 1. Introduction

Biomarkers are naturally occurring molecules which can be indicators of a biological state or condition [1]. Protein biomarkers are particularly useful because of the availability of a large range of analytical instrumentation that can identify and quantify proteins within complex biological samples [2]. Biomarkers can be used clinically to screen, diagnose or monitor the activity of diseases, while also guiding molecular target therapy and measuring therapeutic response [3, 4]. Since the 1960s, biosensors have been researched as analytical devices to convert a biological state into a physiochemical event [3, 4]. Biosensors usually require a biological recognition entity (such as enzymes, antibodies or whole cells [5]), which binds to a chemical target with a high degree of specificity; an ensuing physicochemical signal is then transduced into a measurable output [6]. Biosensors have the advantages of being cost effective and portable tools, allowing for the fast and real time detection of a target analyte, with a high degree of accuracy. However, biosensors which rely on a biorecognition entity have drawbacks. Environmental conditions such as temperature and pH can impair their functions. Denaturation of protein structures can occur outside of the optimum pH and temperature ranges. Such issues when using metastable biological molecules for biorecognition has led to the search for new approaches to replace them using temperature and acid stable synthetic receptors.

Molecularly imprinted polymers (MIPs) form an important class of synthetic bioreceptor that have gained significant attention over the past 30 years. Their unique properties include low cost and facile preparation, high selectivity and sensitivity.

More recently, the molecular imprinting of large biomolecules, such as nucleic acids, viruses and proteins, has become increasingly topical, especially with the aim of developing MIP-based sensors for the detection of disease markers [7, 8]. MIP-based biosensors, have been reported for the determination of a number of protein biomarkers including bovine (and human) serum albumin, bovine haemoglobin (BHb), myoglobin (Mb), cardiac troponin T, ferritin, prostate specific antigen, alpha-fetoprotein, and carcinoembryonic antigen [9–12]. The imprinting of macromolecules and bio-macromolecules is not without challenges, when compared with the imprinting of low molecular weight templates. The molecular imprinting of low molecular weight solutes is by and large conducted in an organic solvent system, the latter chosen to have optimum compatibility with both template and functional monomers [10]. Traditional organic-solvent based MIPs are usually rigid and crystalline and thus lack the polymer chain relaxations that may be required when binding metastable biomacromolecules capable of conformational changes. Protein precipitation or unravelling of the tertiary and/or quaternary conformations of a protein when using an organic solvent [13] can also negatively impacts binding site formation leading to corresponding issues with low affinity and low selectivity of the MIP [14]. The approach with biomolecular imprinting has therefore been to use an aqueous solvent system [15, 16], which allows the biomolecular template to remain structurally stable during and after the imprinting process. The use of water-soluble monomers and cross-linkers in the synthesis of MIPs for biomacromolecular targets is now common place.

The resulting hydrogel materials are hydrophilic and highly crosslinked. Due to their high water compatibility, hydrogel-based MIPs have been shown to retain protein stability and provide a robust means for recognition of target analytes over long periods [17, 18].

**Figure 1.**
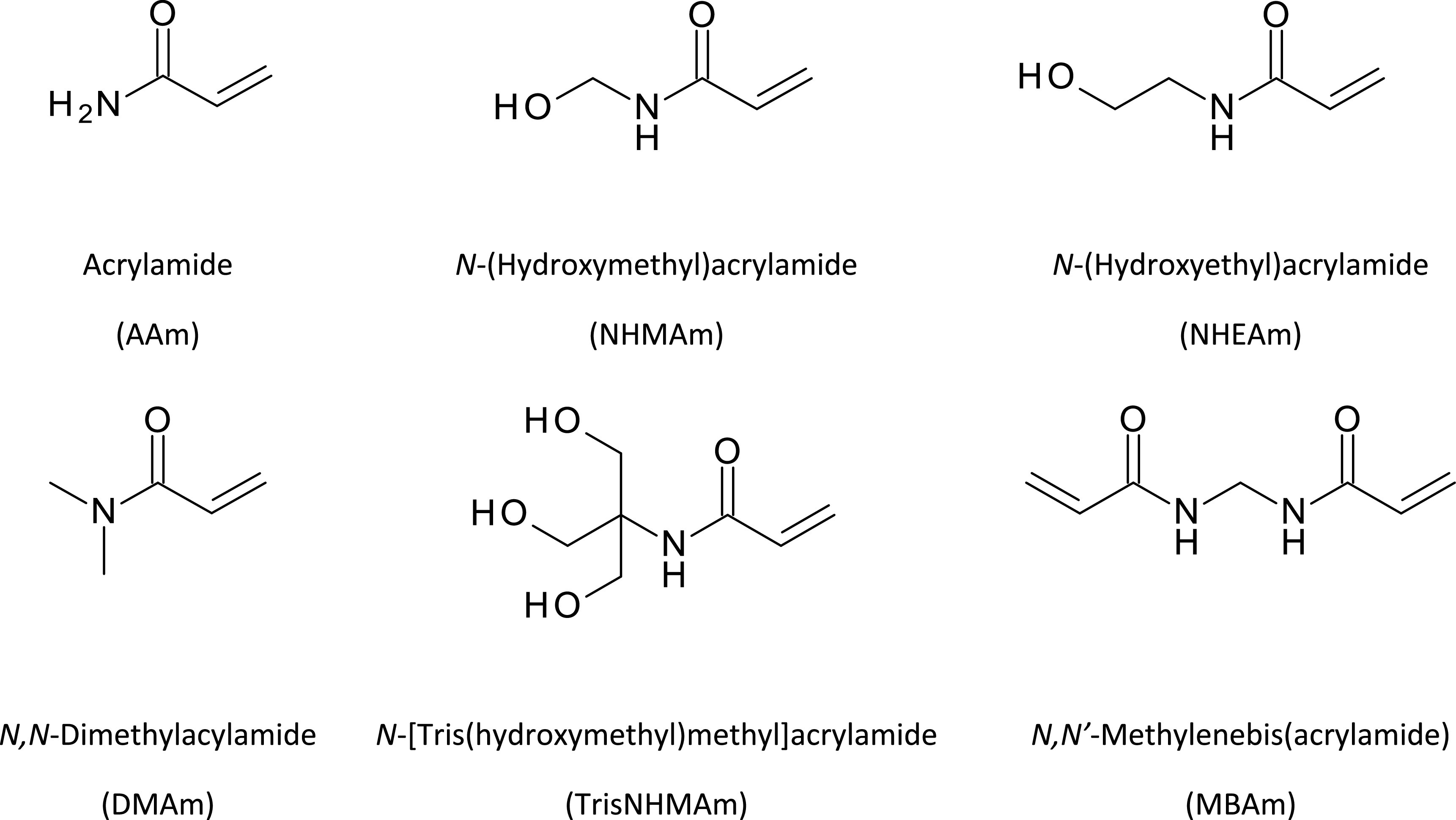
Structural formulae of polymerization mixture components used in the MIP synthesis: the monomers-acrylamide (AAm), *N*-(Hydroxymethyl)acrylamide (NHMAm), *N*-(Hydroxyethyl)acrylamide (NHEAm), *N,N*-Dimethylacrylamide (DMAm), *N*-[Tris(hydroxymethyl)methyl]acrylamide (TrisNHMAm), and the cross-linker-N,N′-methylenebisacrylamide (mBAm).

For hydrogel protein imprinting, water-soluble monomers such as acrylamide and functionalised acrylamides have been used alongside the cross-linker *N,N*′-methylenebisacrylamide (mBAm) to produce polyacrylamide-based hydrogels (figure 1). The amide group of acrylamide monomers can form strong hydrogen bonds. The occurrence of a C=O and C–N dipoles allow acrylamide to act as a hydrogen bond acceptor. Due to the presence of N–H dipoles, acrylamide can additionally act as a hydrogen bond donor [10, 19].

These monomers therefore contain functional groups that are capable of non-covalent (hydrogen bonding and van der Waals’) interactions with the protein template. Hydrogen bonding interactions are essential during the pre-association phase between the template protein and acrylamide monomer [8, 10, 20–23]. Subsequent polymerisation, in the presence of a bi-functional cross-linker can impart the robustness that the polymer requires to retain its form and shape. Once the polymerisation has taken place, the protein has been imprinted.

**Figure 2.**
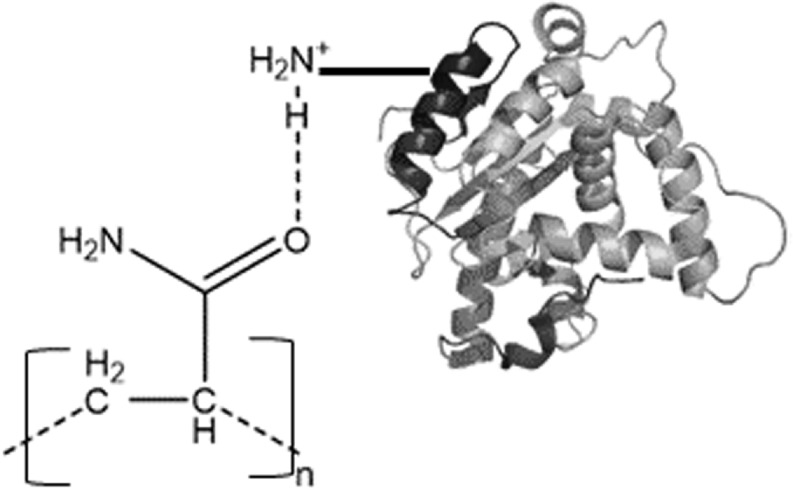
Example of hydrogen bonding that can occur between an acrylamide (AAm) carbonyl (C=O) group and a NH_3_^+^group from a protein (myoglobin) residue.

Hydrogen bonding, can also play a very significant role in the formation of a MIP-protein complex [24–26], The depiction in figure 2 is of the carbonyl group of acrylamide interacting with the quaternary ammonium group typical within a protein [27–29]. Monomers are chosen to optimise such hydrogen bonding effects in order to enhance binding association. Using monomers with hydrogen bonding capability can also result in them being more soluble in water and this is beneficial when preparing protein-based MIPs, thus avoiding denaturation and changes to the conformation of the protein structure if an organic solvent were to be used [10, 18].

In its simplest form, a hydrogel-based MIP can be produced through a one-pot synthesis. An aqueous solution containing functional monomer, template, crosslinker, catalyst and initiator react together at room temperature to form a hydrogel MIP monolith, typically 1–5 ml in volume [10]. This so-called bulk or 3D imprinting method for protein templates creates a hydrogel MIP monolith and has become a common technique [18]. After polymerisation, the monolith is extruded through a sieve, to produce smaller microparticles, and exposing surface bound protein. The protein template is then extracted from the surface of the polymer gel particles through a series of sodium dodecyl sulphate surfactant (SDS) and acetic acid washes. Template removal exposes surface binding sites (so-called cavities) which are capable of recognising and rebinding the same protein [10]. While bulk imprinting is a common method for producing MIPs, it should be noted that the sieve process is not only time-consuming, but also destructive. During gel extrusion through the sieve, the polymer is physically broken into microparticles to expose binding sites on the particle surface. However, this harsh process can potentially damage some of the binding sites themselves, with reports of useable high affinity MIP materials from this process estimated to be around 30%–40% of the total polymer produced [26]. This top-down approach therefore results in significant losses. However, since the bulk MIP approach is more easily scalable and uses low cost monomer reagents, such losses can be acceptable. For example, 3–5g of useable bulk MIP microparticles can be produced per batch within 24 h [10, 30].

Various groups have investigated and progressed bottom-up approaches resulting in nanoparticle-based MIPs. Using similar monomers to the bulk approach, the MIP nanoparticles (typically 100–150 nm in size) are grown with the protein being imprinted on the surface of the nanoparticle [31, 32]. There is therefore no requirement for breaking-up of the polymer (as with the bulk approach) to gain access to and remove the template. Therefore, a greater proportion of the binding sites are retained. However, the reported yields for nanoMIPs are low with the concentration of the nanoMIP solution produced being approximately 100 *μ*g ml^−1^ (for a 100 ml solution) per batch [33].

Alongside bulk (3D) MIP and nanoparticle (NP) MIP approaches, thin film-based (2D) MIPs have also been investigated. These are typically produced on solid substrates such as mica and electrode surfaces. The films (typically 0.1–10 *μ*m thick) are produced either by stamping or spin-coating of a polymerising solution [34], or electrochemical approaches [35]. In all cases, the template binding sites are located on the exposed surface of the thin film. These thin films lend themselves to ready integration to sensor surfaces. However, they are fragile and can degrade during use [36]. Stability can be improved by increasing film thickness, resulting in thin sheets (greater than 200 *μ*m thick) [37].

Various methods that have been investigated to produce a rigidly-coupled thin-film to the sensor surface include electrochemical polymerisation, dip-coating and/or stamping of a polymerising solution [36]. The latter methods typically produce thin MIP films (0.1 to 10 *μ*m). While, such thin-films are crucial to the transduction of a signal with for example electrochemical and quartz crystal microbalance sensing modes [36], there is not necessarily the same restriction on thickness required for optical sensing. Simpler methods of thin-sheet formation for optical sensing applications can be investigated in order to produce thicker and potentially free-standing and transferrable thin sheets.

The aforementioned nanoparticle MIP approaches offer the advantage of higher density of binding sites, compared with either bulk imprinting or thin-sheets. However, the translation of nanoparticle-based MIPs into thin-sheets can present problems due to lack of a dense, homogeneous and continuous layer and challenges of integrating them with a sensor surface [38]. Additionally, the time-consuming and complicated synthesis process makes the nanoparticles unsuitable for large-scale industrial production and application [38].

Herein, we report a method to produce portable MIP thin sheets that can be applied to optical protein sensing. We evaluate thin-sheet hydrogel-based MIPs and compare them against bulk MIPs for protein recognition. We show that thin-sheet MIPs are simple to produce and allow for easy accessibility to template-selective binding sites where binding can occur at or near the surface of the polymer without the need for any post-synthesis processing of the MIP. This is in contrast to the aforementioned bulk imprinting approach requiring aggressive post-processing reducing selective protein binding ability [10, 34, 39, 40].

## 2. Materials

Acrylamide (AAm), ammonium persulphate (APS), bovine haemoglobin (BHb), fetal bovine serum (fbs), hydrochloric acid, glacial acetic acid (AcOH), lysozyme (Lys; from chicken egg white), myoglobin (Mb; from equine skeletal muscle), *N*-(Hydroxymethyl)acrylamide (NHMAm), *N*-(Hydroxyethyl)acrylamide (NHEAm), *N,N*-Dimethylacylamide (DMAm), *N, N*′-methylenebisacrylamide (mBAm), *N*-[Tris(hydroxymethyl)methyl]acrylamide (TrisNHMAm), phosphate buffered saline (PBS), sodium dodecyl sulphate (SDS), sodium hydroxide and tetramethylethyldiamide (TEMED), were all purchased and used without purification from Sigma-Aldrich, Poole, Dorset, UK.

## 3. Methods

*Solution preparations*. A solution of 10% (w/v):10% (v/v) SDS:AcOH was prepared for use in the washing (protein elution) stages before the template reloading stage. SDS (10 g) and AcOH (10 ml) was dissolved in 990 ml of deionised (DI) water, to produce 1 l of the elution solution.

**Figure 3.**
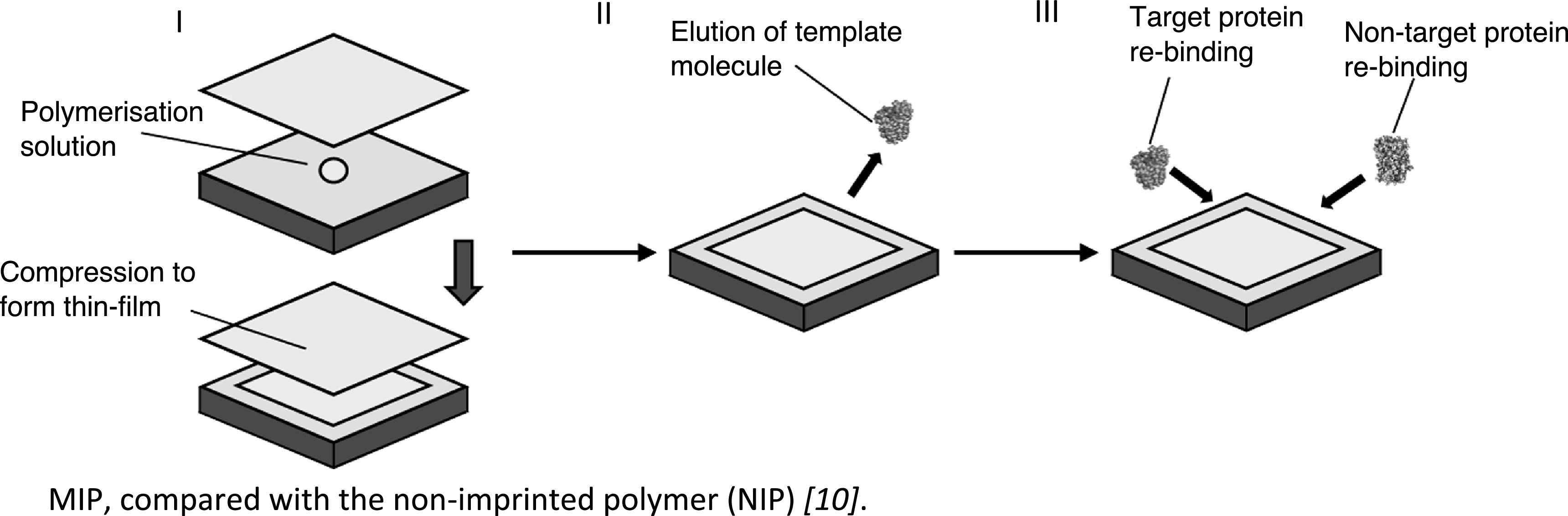
Schematic representation of the synthesis of the thin-sheet (2D) MIP: (I) Template contained polymerising solution placed on to Parafilm^®^, followed by compression using bronze weights, then polymerisation, to form thin-sheets. (II) Elution of templates molecule with 10% (w/v):10% (v/v) SDS:AcOH eluent, for two hours, followed by five water washes . (III) The reloading of target molecule and/or non-specific target to the MIPs.

*MIP preparation.* Thin-sheet MIP hydrogels were produced (figure 3), using an optimised methodology [10], where a 10% cross-linking monomer/N,N′-methylenebisacrylamide hydrogel was found to produce the optimal imprint for Mb, in terms of specificity and rebinding efficiency of the MIP, compared with the non-imprinted polymer (NIP) [10].

The thin-sheet MIPs were produced with different monomers (AAm, NHMAm, NHEAm, DMAm, TrisNHMAm) and a 10% cross-linking density for the protein myoglobin as a template using the following method. Into an Eppendorf tube, 12 mg of myoglobin template was dissolved in 970 *μ*l of deionised water vortexed for 1 min, followed by the addition of 7.6 × 10^–4^ mol of functional monomer and mBAm (cross-linker) at a ratio of 9:1 by weight (individual masses of each monomer and cross-linker are shown in table 1), then vortexed for a further minute. Finally, 10 *μ*l of a 5% TEMED (v/v) solution and 20 *μ*l 5% APS (w/v) solution were added and the mixture was vortexed for 1 min The solution was then poured onto 4 cm^2^ of Parafilm^®^ and covered with another Parafilm^®^ square cutting, before sandwiching between two coverslips. A pressure of 2 kPa was applied using bronze weights and the solution was left to polymerise overnight [40]. Corresponding NIPs were produced using the same method as above but in the absence of a protein template.

**Table 1.** The masses (mg) of monomers, cross-linker (mBAm) and volume (*μ*l) of H_2_O used in the synthesis of the thin-sheet MIPs.

Monomer	Monomer mass (mg)	Cross-linker (mBAm) mass (mg)	Volume of H_2_O (*μ*l)
AAm	54	6	970
NHMAm	77	9	970
NHEAm	87	10	970
DMAm	75	8	970
TrisNHMAm	133	15	970

After polymerisation, the free-standing thin sheet gels (0.4 mm) were cut into circular disks of 120 mg and 90 mm in diameter. The thin sheet gel thickness was determined using a Mitutoyo 500–162–20 Absolute Digimatic Caliper with a 0.01 mm resolution and a ±0.02 mm accuracy. The determination of the thickness is an average of measurements taken at 5 different points of the thin-sheet, repeated 3 times. The disks were then washed with five 1 ml deionised water, followed by soaking in a 1 ml volume of 10% (w/v):10% (v/v) SDS:AcOH eluent, for two hours; this allowed for the removal of the template protein from the MIP cavities. Following this, the gels were washed with five 1 ml volumes of deionised water to remove all residual 10% (w/v):10% (v/v) SDS:AcOH from the thin-sheet MIP gels. Corresponding non-imprinted polymers (NIPs) were synthesized, using the same procedure as the MIPs, but in the absence of the template molecule.

*MIP Rebinding Studies.* The subsequent rebinding effect of the conditioned and equilibrated MIPs and NIPs were characterized using the BioDrop *μ*LITE UV/visible spectrometer. The thin-sheet hydrogels MIPs (120 mg) were placed into an Eppendorf containing 0.72 mg of myoglobin (template protein) dissolved into 1 ml of deionized water (DI). The polymer/protein solutions were left for two hours and allowing to protein rebinding to occur at room temperature (22 ± 2 °C). The polymer was then washed four times with 1 ml of deionized water. The selectivity of the conditioned and equilibrated MIPs was also investigated by immersing the thin-sheet hydrogel MIPs (120 mg) into an Eppendorf containing 0.72 mg of lysozyme (non-target protein) dissolved into 1 ml of deionized water (DI).

*Computational Details.* Quantum mechanics (QM) calculations of C=O vibrational frequency shifts for simplified models (as described in the results), were calculated using density functional theory (DFT) with M05–2X [41] and the 6–31 + G** basis set [42, 43], given its performance for description of non-covalent systems [44]. All geometries were optimized and validated as minima with all real frequencies. Vibrational frequencies were scaled using the recommended scaling factor (0.936) [45] for the M05–2X/6–31 + G** method. All DFT computations were performed with Jaguar 9.2.

*FTIR Spectroscopy Characterization.* MIP and NIP thin-sheets were analysed using an Agilent Cary 620 Fourier transform infrared spectrometer interface with an Agilent Cary 620 microscope fitted with a 15×-cassegrain objective and a narrow-band liquid nitrogen cooled detector. The microscope was operated in the reflectance mode, with each sample run at 16 scans, with a scan range of 400–4000 cm^−1^, an aperture source of 2 cm^−1^ at 4000 cm^−1^, with the spectral resolution at 2 cm^−1^ and a beam attenuation throughput of 50%. In all cases the incident infrared beam was focused at a thin-sheet of MIP layered upon a gold coated disk. Agilent Resolution Pro software was used to analyse the spectra.

## 4. Results and discussion

Here we evaluate bulk to thin-sheet hydrogel-based MIPs for protein recognition. Having previously developed a series of polyacrylamide-based hydrogels MIPs for protein recognition, we hereby build upon this with the creation of a series free-standing thin-sheet polyacrylamide hydrogel MIPs, for the specific selection of the target protein myoglobin. These thin-sheet MIPs potentially offer the same robustness and high performance as our previous MIPs, but without the need for laborious grinding or sieving. The selectivity of the MIPs was investigated with the non-target protein lysozyme , because of its similarity in size (myoglobin (17.0 kDa) and lysozyme (14.3 kDa)) . Computational studies were undertaken to predict whether the hydrogen bonding effects caused by the target protein (myoglobin) binding within a MIP cavity could cause any peak shifting within the infrared region. This peak shifting was investigated using FTIR spectroscopy.

### 4.1 Rebinding experiments

We produced a series of acrylamide-based (AAm, NHMAm, NHEAm, DMAm, and TrisNHMAm) hydrogel MIPs with selective recognition for the target protein myoglobin [30]. By comparing the MIPs with their corresponding non-imprinted polymer (NIP) controls, an imprinting factor (IF) was calculated, using equation (1) and used to assess performance. We explored the selectivity of these MIPs further by studying their binding with the non-target protein, lysozyme, chosen due to similarity in size and hydrophobic solvent accessible surface areas (SASA), allowing for the calculation of the selectivity factor (SF) using equation (2).

\begin{eqnarray*}IF=\displaystyle \frac{ \% \,protein\,rebind\,to\,MIP}{ \% \,protein\,bind\,to\,NIP}\end{eqnarray*}

\begin{eqnarray*}SF=\frac{ \% \,target\,protein\,\left(mb\right)\,rebind\,to\,MIP}{ \% \,\,non-target\,protein\,\left(lys\right)\,bind\,to\,MIP}\end{eqnarray*}

The IF was calculated as a ratio of the amount of target rebound to the MIP to the amount of target protein that bound to the corresponding NIP. IF is commonly used to evaluate the imprinting effect and is a measure of the strength of interaction between the functional monomer and the target molecule. The higher the IF value, the more selective the MIP is for the target molecule, with an IF > 1.20 generally considered favourable. A selectivity factor (ratio of MIP binding to target versus a non-target protein) is generally accepted as a better determinant of selectivity and again an SF > 1.20 is now generally considered more favourable [46–48]. This is shown in table 2.

**Table 2.** Percentage of the myoglobin target protein rebind to the five different acrylamide-based MIPs and NIPs, and their corresponding impact factors. Results for co-monomer combinations A (TrisNHAm + DMAm) and B (NHEAm + DMAm) are also shown.

Monomer/Co-monomer	MIP percentage of myoglobin rebind (%)	NIP percentage of protein bind (%)	MIP percentage of lysozyme rebind (%)	Imprinting factor (IF)	Selectivity factor (SF)
NHMAm	98.9 ± 0.2	51.8 ± 0.4	41.5 ± 3.7	1.90	2.38
AAm	85.4 ± 1.0	47.5 ± 4.2	42.4 ± 1.8	1.80	2.01
NHEAm	77.2 ± 3.0	43.6 ± 1.3	44.3 ± 2.1	1.77	1.74
TrisNHMAm	79.9 ± 4.8	72.3 ± 1.7	59.7 ± 3.1	1.10	1.34
DMAm	72.0 ± 3.0	48.8 ± 0.9	43.2 ± 1.9	1.48	1.67

The generally accepted, self-assembly method to produce MIPs, relies on there being an initial degree of association between the monomer and the template in solution, and mainly depends on hydrogen-bonding interactions [49]. Therefore, the monomers that contain hydroxy groups (NHMAm, NHEAm and TrisNHMAm), have the potential for stronger binding towards the template, in contrast to the monomers AAm and DMAm. The non-selective binding by the NIPs also supports this, where the –OH containing monomers, TrisNHMAm and NHMAm, demonstrate the highest amount of protein binding (72.3% and 51.8%, respectively), whereas the AAm NHEAm NIP demonstrate lower values of 47.5% and 43.6%, respectively. However, when the monomers are ranked by the percentage of protein that rebinds to the MIP (NHMAm > AAm > TrisNHMAm > NHEAm > DMAm), the MIPs do not follow the same trend as the NIPs. Of note, the monomer AAm, although void of –OH groups, is ranked second, and superior to both NHEAm and TrisNHMAm. Additionally, TrisNHMAm, although possessing three –OH groups capable of hydrogen bonding, it rebinds target only marginally better than the more hydrophobic NHEAm (77.2%) and DMAm (72.0%). While this would seem somewhat surprising, work by Kryscio *et al* [14] showed that pre-polymerisation protein-monomer complexes can potentially cause changes to the secondary structure of the protein and force proteins into various conformations and aggregates that if imprinted, can produce MIPs which lack the desired target selectivity. Monomers interacting with proteins could disrupt the secondary structure within the protein, especially if the monomer interactions are with functional groups along the protein backbone [50]. Indeed, Sullivan *et al* [30] showed that the hydroxyl groups in TrisNHMAm were able to strongly hydrogen bond to helical backbone residues within the potential binding sites of myoglobin leading to changes in this target’s secondary structure. In addition to this, TrisNHMAm has a low IF value of 1.10 suggesting that the MIP behaves similarly to the NIP and is therefore deemed unacceptable for use as a MIP. Therefore, other factors besides the potential strength of protein-template interactions, need to be considered when choosing a suitable monomer for a target protein. With regards to selectivity NHMAm and AAm still outperform the other monomers with SF values of 2.38 and 2.01, respectively. While, the selectivity factor (1.34) of the TrisNHMAm MIP is improved when compared to the corresponding imprinting factor 1.10, this MIP still has the lowest selectivity. This further supports that the TrisNHMAm monomer is disrupting the secondary structure of the template protein within the prepolymerization mixture, hence producing a MIP less selective for the target.

The five acrylamide-based monomers (AAm, NHMAm, NHEAm, DMAm, and TrisNHMAm) were further studied to produce thin-sheet MIPs and NIPs, using myoglobin as the template/target. These monomers and target proteins were chosen due to our previous success with them in bulk imprinting [30]. The thin-sheets were cut into uniform circular disks of 120 mg and 90 mm in diameter. Template removal and rebinding protocols developed by Hawkins *et al* [10] were used in this study. After the template protein (myoglobin) was removed from the disks using a 10% (w/v):10% (v/v) SDS:AcOH, the disks were then place into Eppendorf tubes with 0.72 mg of protein, either myoglobin (target protein) or lysozyme (non-target protein), dissolved into 1 ml of deionized water. The binding of the target protein myoglobin was performed on the thin-sheet MIPs and their corresponding NIPs to determine the percentage of the target protein able to rebind. The binding of the non-target protein lysozyme was also studied, in order to determine the selectivity of the MIP. The subsequent imprinting factors (IFs) and selectivity factors (SFs) were again calculated using equations (1) and (2), respectively.

**Figure 4.**
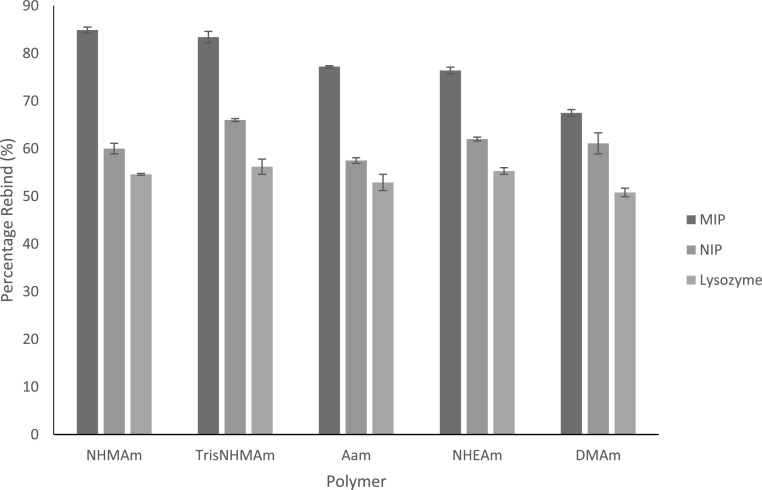
Percentage of the myoglobin target protein and lysozyme non-target protein rebind to the five different acrylamide based thin-sheet MIPs and NIPs.

The rebinding studies are presented in figure 4 and table 3 and show that the NHMAm polymer produced the thin-sheet with the highest myoglobin rebinding percentage and thus having the greatest target protein recognition ability, with 84.9% of the myoglobin being rebound to the MIP, an imprinting factor of 1.41 and a selectivity factor of 1.55. The thin-sheet MIP with the lowest myoglobin rebinding percentage was DMAm, with only 67.5% of the protein myoglobin being able to rebind and having a corresponding imprinting factor of 1.11 and selectivity factor of 1.32. With the exception of DMAm, all the MIPs possessed an IF value above 1.2 and would therefore generally be considered to have good recognition for the target/template molecule. This is comparable with the work of Zayat *et al* which produced similar imprinting factors of 1.4 for polyacrylamide thin-sheets (of similar monomer and crosslinker density) imprinted for the target, maltose binding protein [48]. This shows that the thin-sheet MIPs that have been produced in this work using a range of functionalised acrylamide monomers, are able to offer good selectivity for the target protein. With respect to overall MIP efficacy, NHMAm > TrisNHMAm > AAm > NHEAm > DMAm. This is consistent with our previous bulk MIP analysis, which also shows the monomers NHMAm and DMAm, to be the highest and lowest performing monomers, respectively [30]. While the percentage rebinding for the thin-sheet MIPs, generally follow the same pattern as the bulk MIPs [30], there is one notable exception, TrisNHMAm performs much better as a thin-sheet MIP. When the non-target protein lysozyme was loaded onto the MIPs, the percentage of the protein that bound was similar ranging from the lowest, DMAm at 50.8% to the highest TrisNHMAm at 56.2%. This provided selectivity factors all greater than 1.32, showing that all the MIPs were selective for the target protein myoglobin and not other proteins. This is consistent with the work of Matsunaga *et al* who immobilised an acyclic acid-based (crosslinked with MBAm) thin-sheet MIP onto a surface plasma resonance (SPR) chip surface, for the detection of lysozyme [51]. In this work cytochrome *c* was loaded onto the MIP and measured using SPR, in order to determine selectivity of the MIP. The calculated selectivity factor for this MIP was 1.2, which agrees with the selectivity of our MIP (with SF > 1.32). With respect to our previous bulk MIPs, the performance of these new thin-sheet MIPs is slightly reduced [30], but not enough to discount their applicability. For example, the percentage rebind for the bulk NHMAm MIP is 98.9% (SF 2.38) compared with the thin-sheet NHMAm MIP is 84.9% (SF 1.55). This shows that the performance of the thin-sheet MIP is comparable with previous work and literature, with the additional benefits of simplistic synthesis, processing and usability of the thin-sheet [30, 51]. Moreover, when tested with a non-target protein (lysozyme) the MIPs where shown to be highly selective against the non-target, again with SF comparable with literature [51]. It should also be noted that the bulk (3D) MIP possesses a much greater surface area : volume ratio and therefore a higher density of template active binding sites compared with the thin sheet (2D) MIPs.

**Table 3.** Percentage of the myoglobin target protein and lysozyme non-target protein rebind to the five different acrylamide based thin-sheet MIPs and NIPs, and their corresponding impact factors and selectivity factors.

Monomer/Co-monomer	MIP percentage of Mb rebind (%)	NIP percentage of protein bind (%)	MIP percentage of lys bind (%)	Imprinting factor (IF)	Selectivity factor (SF)
NHMAm	84.9 ± 0.6	60.0 ± 1.1	54.6 ± 0.2	1.41	1.55
TrisNHMAm	83.4 ± 1.2	66.0 ± 0.3	56.2 ± 1.6	1.26	1.48
AAm	77.2 ± 0.2	57.5 ± 0.6	52.9 ± 1.7	1.34	1.46
NHEAm	76.4 ± 0.7	62.0 ± 0.4	55.3 ± 0.7	1.23	1.38
DMAm	67.5 ± 0.7	61.1 ± 3.2	50.8 ± 0.9	1.11	1.32

**Figure 5.**
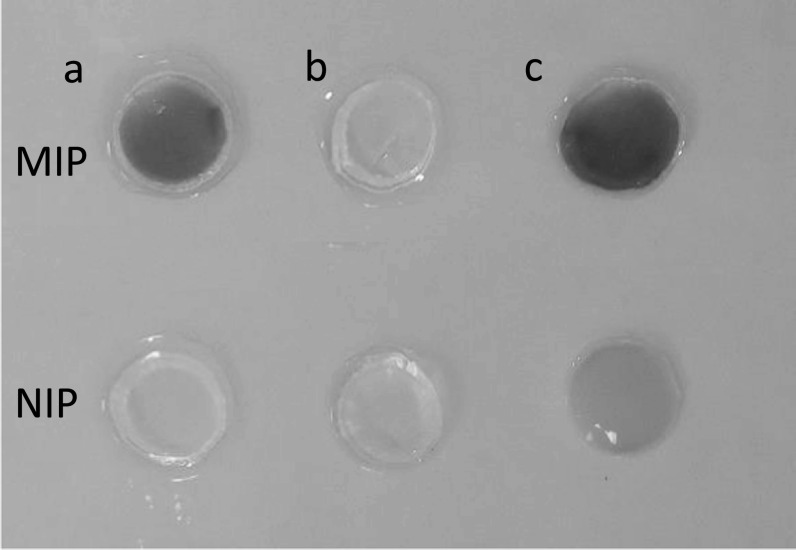
Optical images showing the removal and rebinding of myoglobin target protein: (a) freshly prepared MIP/NIP with target protein still loaded, (b) MIP/NIP with the target protein eluted, (c) MIP/NIP after the target protein has been reloaded.

Myoglobin being a coloured protein allowed us to use optical inspection as an additional and simple method to confirm protein removal and rebinding within a MIP and NIP (figure 5). While such optical inspection is possible with coloured proteins, in order to also assess colourless proteins, we would need to refer to for example UV/Visible spectroscopic techniques and evaluate the absorbance at 280 nm (A_280_) to quantitatively and qualitatively assess protein removal and rebinding within the MIP [47].

The disadvantage here is that we are assaying the unbound protein in solution and subsequently determining the amount of protein bound to the MIP; we are therefore not directly interrogating the MIP for protein binding. The UV/Visible spectrometer does not readily lend itself to the direct interrogation of thin sheets. Due to its method of operation, the degree of light absorbed and transmitted through the sheet will vary in a non-linear fashion depending on changing thickness (due to possible swelling/deswelling) and refractive index of the thin-sheet MIP. FTIR spectroscopy has been used to explore hydrogen bonding within molecules. Hydrogen bonding can cause the vibrational bands within spectra to vibrate at lower frequencies, as long as these vibrational bands are actively involved and participating in hydrogen bonding [52, 53]. Where vibrational bands are not associated with hydrogen bonding, FTIR spectral bands remain unaffected [54]. We surmise that FTIR spectroscopy of imprinted polymers may therefore reveal information on the protein binding state of MIPs. More specifically, hydrogen bonding may cause the vibrational modes in polar bonds to vibrate at lower frequencies than before. We therefore investigated ATR-FTIR spectroscopy to interrogate protein binding only to the surface layers of the thin sheet MIP. Specifically, we investigated changes to the C=O stretch of the acrylamide MIPs and NIPs as a function of protein binding in order to elucidate a hydrogen bonding signature for selective protein binding to MIP.

### 4.2 Computational calculations

It is understood that only when the cognate protein is selectively docked into the corresponding MIP, the strong hydrogen bond donor residues (for example, lysine), within the protein, are responsible for the hydrogen bonding related shift in carbonyl frequency. As a simple test, using DFT at the M05–2X/6–311 + G** level of theory [41–43], the shift in the C=O vibration frequency of acrylamide, was investigated. An optimized complex of AAm hydrogen, bonded with a single water molecule was compared with AAm binding to a model (NH_3_^+^−CH_3_) of +1 charged lysine. The lowering of the frequency was observed when switching from water (hydrogen bond distance = 1.9 Å) to the lysine model (hydrogen bond distance = 1.6 Å), by 30 cm^−1^.

### 4.3 FTIR Characterization

The free-standing hydrogel MIP and NIP sheets produced in this study provided uniform and homogeneous layers, with a thickness of 0.40 ± 0.03 mm. FTIR spectroscopy was used to characterize these sheets, using the reflection mode to probe the polymer surface. This uses true specular reflectance as a surface measurement technique, working on the principle of reflective efficiencies, with every sample having a refractive index, which varies with the frequency of light to which it is exposed. Instead of examining the energy that passed through the sample, true specular reflectance measures the energy that is reflected off the surface of a sample or its refractive index [55]. This technique is most suited to the thin smooth layer of the MIPs and NIPs, with the thin smooth layer allowing the incident light to reflect from the surface of the metallic plate and exit from the surface of the sample.

The FTIR spectra of the myoglobin imprinted MIPs hydrogel thin-sheets are shown in figure S1A (available online at stacks.iop.org/BPEX/7/045025/mmedia), and are characteristic of polyacrylamide-based hydrogels, with a high-water content (94% water). The strong broad peaks at approximately 3400 cm^−1^ can be assigned to O–H stretching of water, and the small sharp peaks approximately 2950 cm^−1^ can be assigned to C-H stretching within the polymer. The strong broad peaks between the range of 1600–1700 cm^−1^, an enhancement of S1A (between 2000–1000 cm^−1^) shown in figure S1B, are assigned to the C=O stretching within the polymer hydrogels. These peaks are interestingly broader than expected, and this is due to the hydrogen bonding effects caused by the water molecules within the hydrogels. There is an absence of N–H stretching peaks (approximately 3300–3400 cm^−1^), which could be possibly due to the high percentage (94%) of water present, resulting in the weak N–H stretching peaks, usually seen, being masked by the strong O–H stretching peak of the water molecule. Furthermore, the C=O stretching peak (1600–1700 cm^−1^) being much broader than usually seen, could possibly explain the absence of the weak and sharp N–H bending peaks that would be seen in the same areas. The broad medium peak seen at around 1000 cm^−1^ is assigned to the C–N stretching peak, again this is broader than expected and is possibly due to the hydrogen bonding effects caused by water molecules within the hydrogel. It should be noted that the amide I and amide II bands, from secondary structures [56] of the target protein, are not readily observed in the FTIR spectrum of the protein bound MIP, since these bands are masked by the stronger carbonyl peak of the polyacrylamide hydrogel. Figure S2 is the FTIR spectrum for the non-imprinted polymer (NIP), and shows the same characteristic peaks as the MIPs in figure S1(A), proving that the NIPs and MIPs are of the same polymeric material, with figure S2(B) being an enhancement (between 2000–1000 cm^−1^) of figure S2(A), again focussing on the C=O stretching vibrational peaks, in the range of 1600–1700 cm^−1^, within the polyacrylamide-based hydrogels. The C=O peak shifts, for the five thin-sheet MIPs (AAm, NHMAm, NHEAm, DMAm, and TrisNHMAm), at their different protein bound states, are summarised in table 4.

**Table 4.** The carbonyl (C=O) amide peaks for different monomer thin-sheet MIPs and NIPs at the different protein bound states.^a,b^

	Peak (cm^−1^)				
MIP/NIP status	AAm	NHMAm	NHEAm	DMAm	TrisNHMAm
Fresh NIP	1698	1621	1693	1696	1692
NIP washed	1698	1621	1693	1696	1692
Target reloaded (Mb)	1698	1621	1693	1696	1692
Target reloaded from serum (Mb)	1698	1621	1693	1696	1692
Fresh MIP (Mb intact)	***1694***	***1610***	***1685***	1696	1692
MIP washed (Mb eluted)	1698	1621	1693	1696	1692
Target reloaded (Mb)	***1694***	***1610***	***1685***	1696	1692
Non-target loaded (Lys)	1698	1621	1693	1696	1692
Non-target loaded (BHb)	1698	1621	1693	1696	1692
Target reloaded from serum (Mb)	***1694***	***1610***	***1685***	1696	1692

^3^ Carbonyl amide peaks that demonstrate a peak shift associated with protein binding are highlighted in ***bold italics***.

^4^ All peak values are the result of 3 consecutive measurements across the thin-sheet surface.

The NIPs did not exhibit any shift in the carbonyl peak when target protein was loaded onto the polymer. This is be expected as NIPs lack template-specific cavities or binding sites, meaning any resulting binding displayed by the NIP is not from carbonyl-specific hydrogen bonding. As the NIP is a hydrogel containing 94% water, there is a potential for the protein template to be absorbed within the polymer matrix. When the MIPs, AAm, NHMAm, and NHEAm are bound with target protein (Fresh MIP (Mb intact) and target reloaded (Mb)) the carbonyl peak is at the lower values of 1694, 1610 and 1685 cm^−1^, respectively, compared to when target protein is not bound (MIP washed (Mb eluted), non-target loaded (Lys) (figure S3) and non-target loaded (BHb) (figure S4)), 1698, 1621, and 1693 cm^−1^, respectively. This downshift of 4, 11 and 8 cm^−1^ for AAm, NHMAm and NHEAm, respectively, is suggestive of hydrogen bonding effects due to selective target protein binding. However, this downshift value is much lower than expected (compared with computational experiments) and furthermore, the DMAm and TrisNHAm MIPs did not show any downward shift when these MIPs were bound with the target protein. This can be expected with the TrisNHMAm MIP as the functional monomer (TrisNHMAm) in this MIP contains three hydroxyl groups, which are the main functional groups within the MIP cavity, where subsequent binding takes place [30]. As a result, the FTIR peak attributed to the carbonyl peak does not shift, instead we would expect to see a broad FTIR peak in the OH region (approximate wavelength 3000–3500 cm^−1^) due to these hydroxyl groups; as the hydrogel is composed of 94% water (i.e. only 6% polymer material), the TrisNHMAm–OH peaks would be heavily masked by the broad –OH band of water. Additionally, the lower than expected or no downshift seen in the spectra could also be explained by the ratio of protein molecule to monomer molecules being approximately 1 : 1000 in the polymerization mixture. With the vast majority of monomer-protein interactions taking place on the surface of the protein [30], only a small percentage (less than 10%) of the monomers are taking part in hydrogen bonding. To explore the MIPs for their potential use within a biological sample, further testing of MIPs produced using each of the monomers (AAm, NHMAm, NHEAm, DMAm, TrisNHMAm) was conducted in fetal bovine serum spiked with the target protein myoglobin (figure S5). This was repeated for all corresponding NIPs (figure S6). The results show that the MIPs produced from AAm, NHMAm and NHEAm, produced the same peak shifts (4, 11 and 8 cm^−1^, respectively) as the corresponding reloading studies in PBS, while DMAm and TrisNHMAm did not produce any shifts (again, consistent with the previous reloading studies). These biocompatibility results suggests that the target protein can be selectively bound to the MIP from a serum sample that contains a range of different proteins, including bovine serum albumin (BSA) and globulins [57].

FTIR spectroscopy potentially offers a powerful tool to follow selective MIP-protein interactions. However, further work is required to understand the range and breadth of polymers for which it would be applicable.

## 5. Conclusions

Here we have shown the production of simple and easily usable acrylamide-based hydrogel thin-sheet MIPs, which are capable of selectively binding a specific target protein. These thin-sheet materials provide good performance with the NHMAm monomer producing the MIP with optimal rebinding of the target protein and the overall MIP efficacy for the monomers decreasing in the order NHMAm > TrisNHMAm > AAm > NHEAm > DMAm. The thin-sheet MIP performance is comparable with previous bulk MIP work, with the additional benefits of the simplistic synthesis and processing, and transferability. Quantum mechanical calculations (based on a simple model) predicted a noticeable downshift in the FTIR carbonyl peak of the polymer, when target protein is bound to the MIP, caused by hydrogen bonding within the protein-MIP complex. This effect was seen experimentally, with only NHMAm, AAm, and NHEAm based MIPs. The development of these unique thin-sheet hydrogel MIPs offers a simple and effective method to produce a robust biorecognition material in the form of a uniform and free-standing portable layer, that is easily movable from surface to surface. The materials we have produced could easily be adapted for biosensing purposes and potentially incorporated onto the surface of a sensor leading to their use within a portable MIP-based biosensor, leading to the future development of chemical and biosensors that are capable of detecting protein biomarkers.

## Data Availability

All data that support the findings of this study are included within the article (and any supplementary files).

## Conflicts of Interest

There are no conflicts to declare
